# Topological transverse thermoelectrics: A new way forward?

**DOI:** 10.1016/j.xinn.2023.100530

**Published:** 2023-10-20

**Authors:** Shubham Yadav, B.M. Anil Kumar, Satya N. Guin

**Affiliations:** 1 Department of Chemistry, Birla Institute of Technology and Science, Pilani – Hyderabad Campus, Hyderabad 500078, India

## Main text

**Figure 1:**
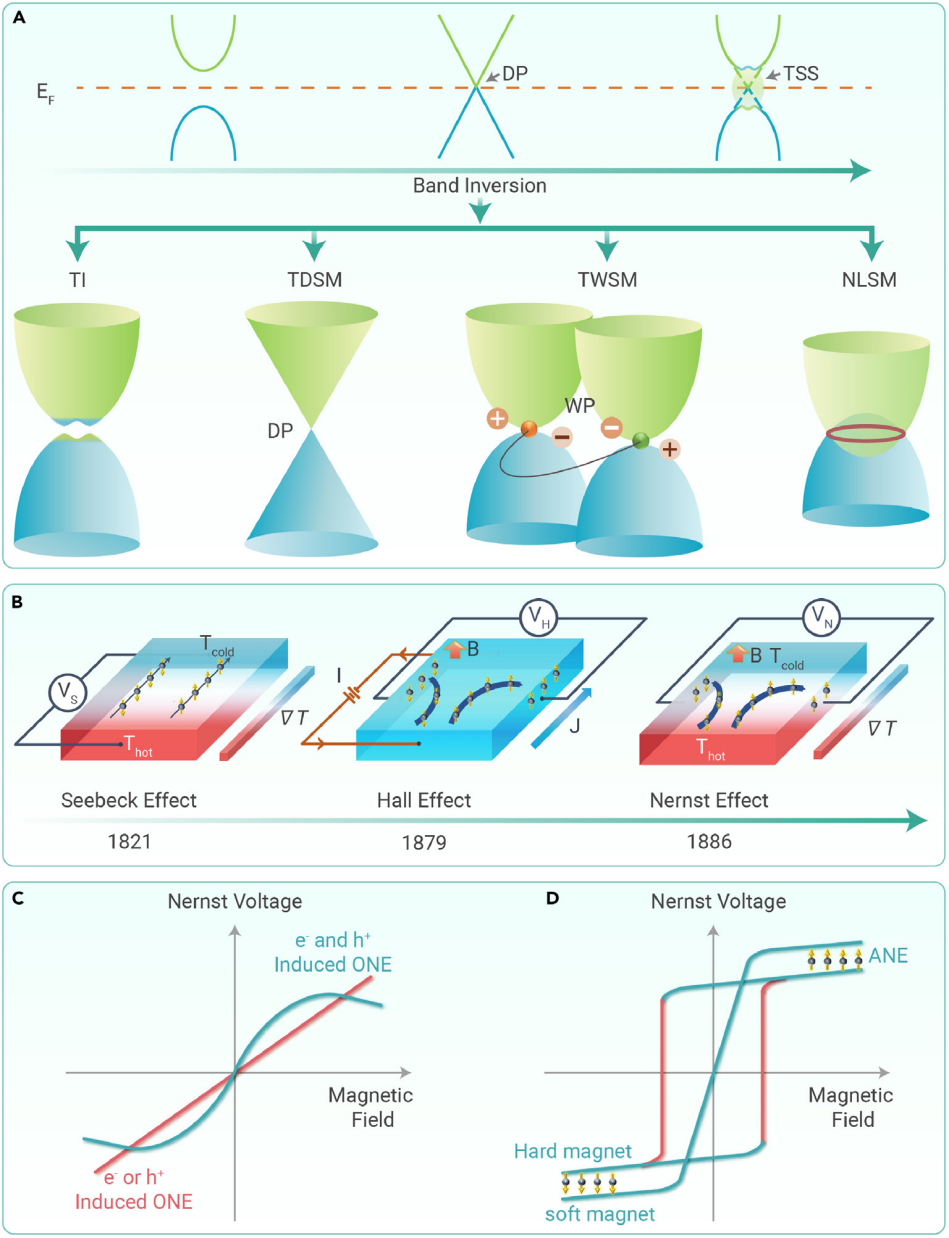
Schematics of different topological states, thermoelectric and Hall measurement configuration, and Nernst signal response curve (A) Representative scheme of band inversion and band structure of different types of topological systems including topological insulators (TIs), topological Dirac semimetal (TDSM), topological Weyl semimetal (TWSM), and nodal line semimetal (NLSM) (from left to right). DP, Dirac point; TSS, topological surface state; WP, Weyl point. (B) Historical development of thermoelectric configurations according to the Seebeck effect, Hall effect, and Nernst effect. (C and D) Magnetic-field-dependent ordinary and anomalous Nernst signal response curves for non-magnetic and magnetic materials, respectively.

Electronic structure is one of the most fundamental factors that decide the physical properties of a material. In the past 18 years, a deeper fundamental understanding of the electronic band structure of material has resulted in the development of topological band theory, which has re-classified solid-state materials into two different classes: topologically trivial and topologically non-trivial. Topologically non-trivial systems provide a new opportunity for material design for fundamental and functional applications from experimental and theoretical viewpoints. As of now, we have witnessed the discovery of different topological systems based on the nature and number of band crossings (Figure 1A). In a nutshell, the linear crossing bands with topological protection and Berry curvature of the non-trivial band open up a new degree of freedom for observing extraordinary physical properties, including high carrier mobility, magnetoresistance, catalysis, anomalous Hall effect, thermoelectrics, etc.^1^^,^^2^^,^^3^

Heat is one of the major “waste” products after the use of different forms of energy. Thermoelectricity, the conversion of “waste” heat into electric energy, is considered a future technology for energy harvesting for practical applications. The heat-to-electricity conversion efficiency of a thermoelectric material is related to the dimensionless thermoelectric figure of merit, $zT=\frac{\sigma S^{2}T}{\kappa }$, where σ, κ, *S,* and *T* are electrical conductivity, thermal conductivity, thermopower, and temperature, respectively.^2^^,^^3^^,^^4^^,^^5^ The field of thermoelectrics has been well known since the early 19th century, with subsequent developments in different thermal, electrical, and thermomagnetic effects, such as the Seebeck effect, Hall effect, and Nernst effect, respectively, as presented in Figure 1B. Although it had been known for a long time, tremendous growth in research activity in thermoelectrics occurred in the late 20th century and in the recent past because of the urgent need for energy conversion devices and sustainability. In the past several decades, the development of high-performance thermoelectric materials has been accomplished using degenerate semiconductors based on the traditional approach (i.e., the Seebeck effect). Traditional thermoelectric research achieved many important conceptual developments, such as band convergence, resonant level, solid solution alloying, nanostructuring, and the quantum confinement effect for the improvement of thermoelectric performance.^5^ On the basis of these approaches, numerous material design and discoveries have been made in the past 20 years. Nowadays, thermoelectric generators (e.g., Bi_2_Te_3_-based modules) are readily available in the market. However, challenges and improvements are required, including interface engineering, compatibility factor, device lifetime, and mechanical strength necessary for new-generation materials.^5^ To eliminate such challenges, transverse thermoelectrics based on the Nernst effect (i.e., the generation of an electric signal perpendicular to both an applied temperature gradient and the external magnetic field) is receiving increasing attention.^2^^,^^3^^,^^4^ The Nernst effect is the thermal analog of the Hall effect, and the Nernst signal response curve in a material resembles to Hall effect response curve. This effect was originally discovered in 1886 in elemental bismuth, and thereafter many superconductors, including heavy fermion, were investigated. The advantages of this device geometry include no requirement for both *p*- and *n*-type materials and electrical contacts’ being made only on the cold side, which can avoid the interface reaction.^2^^,^^3^^,^^4^ However, this was not investigated rigorously, like the Seebeck effect for energy conversion applications. This might be due to the requirement for a magnetic field for its operation and a large focus of research on the Seebeck effect. The development of permanent magnets and observation of zero-field Nernst signals created a new scope. Moreover, in recent years, the promise delivered by topological materials in different areas for functional applications provides an additional opportunity for researchers. The topologically non-trivial band-dominated non-zero Berry curvature effect offers a large Nernst effect in topological materials.

As displayed in Figure 1C, if a particular type of charge carrier dominates, then the Nernst thermopower as a function of the magnetic field shows linear behavior. This is known as the ordinary Nernst effect (ONE). In many materials, particularly in semimetals, multiple valence/conduction bands can cross the Fermi energy. In such materials, comparable amounts of both the electron (e^−^) and hole (h^+^) charge carriers can exist at once. When this happens, the magnetic-field-dependent ONE is no longer linear (Figure 1C). For magnetic materials, the Nernst response curve is similar to field-dependent magnetization or Hall resistivity curve (Figure 1D). This type Nernst response curve is known as the anomalous Nernst effect (ANE). The general magnetization scaling relation of anomalous Nernst thermopower, |$S_{xy}^{A}$| = |$N_{xy}^{A}$|μ_0_*M*, where *M* is magnetization and $N_{xy}^{A}$ is the anomalous Nernst coefficient, is not valid for topological ferromagnets. Therefore, a giant ANE response can be achieved compared with trivial magnetic material with comparable magnetic moment value. Moreover, in magnetic topological systems, there is a rapid rise of signal in a low or zero magnetic field region, which makes them more appealing for practical application.

One of the earliest discovered topological systems, Weyl semimetal NbP, displays a large ONE in both single- and poly-crystalline forms. Many other non-magnetic topological Dirac and Weyl semimetals have been investigated, such as Cd_3_As_2_, ZrTe_5_, Pb_1-x_Sn_x_Te, WTe_2_, etc., and the search is ongoing.^2^^,^^3^^,^^4^ Apart from this, there has been development in magnetic materials for ANE because of the requirement for a lower or zero magnetic field. Examples of few important topological materials include Co_2_MnGa, Co_3_Sn_2_S_2_, Fe_3_Sn_2_, YMn_6_Sn_6_, YbMnBi_2_, Fe_3_(Al/Ga), Mn_3_Sn, Mn_3_Ge, etc.^2^^,^^3^^,^^4^ The topological magnets exhibit higher ANE value than expected from magnetization scaling relation, but it is still much lower than state-of-the-art thermoelectric materials.

In summary, the Nernst measurement of topological materials is a new avenue for understanding topological band features and applications in thermoelectrics. To date, commendable progress has been made, considering the short period of development in the field. Although transverse thermoelectrics have multiple advantages, they must travel a long path, as many challenges must be addressed for their implementation in practical devices. These can be summarized as follows.

### Low thermal conductivity topological materials for high conversion efficiency

The overall conversion efficiency of a Nernst thermoelectric device is related not only to Nernst thermopower but also to thermal conductivity. Therefore, it is equally important to look for topological materials that show high Nernst thermopower and low thermal conductivity. Theoretically highly dispersive bands, small Fermi surface, and small Fermi arcs are important for the observation of high mobility and a large Nernst effect. On the other hand, materials with low phonon group velocity are important for the observation of low lattice thermal conductivity. In this regard, the investigation of both electronic and phonon structures of topological systems will play a significant role in the identification of suitable candidates for experimental realization.

### Observation of large Nernst signal above room temperature

Most of the materials studied to date exhibit large signals below room temperature. However, for the practical application of transverse thermoelectrics for power generation application, it is important to look for new materials. In this context, materials with high magnetic transition temperatures will play a major role. Apart from the intrinsic topological effect, the additional extrinsic contribution can also be useful for enhancing the Nernst signal. In this direction, it has recently been observed that interactions between magnons and electrons in a high spin-orbit coupling (SOC) magnetic system can lead to larger transverse thermoelectric effect because of extrinsic magnon-electron spin-angular momentum transfer. Therefore, magnetic systems with high Curie temperatures and large SOC will be excellent candidates for studying magnon-mediated transport properties, hence providing routes for the enhancement of large transverse thermoelectric response. Another potential class of materials would be goniopolar materials. These materials exhibit an axis-dependent conduction polarity that was recently identified as promising candidates for transverse thermoelectric performance. The intrinsic topological features of the Fermi surface (e.g., NaSn_2_As_2_) or multi-carrier systems (e.g., Re_4_Si_7_) with unequal carrier mobility result in strong axially dependent conduction polarity across different crystallographic directions.^4^

### Requirement for a magnetic field

One of the major impediments with Nernst devices is the requirement for a magnetic field. Such a problem can be tackled with a permanent magnet or hard magnet system. Therefore, a permanently magnetized system can be used for device without the requirement for a magnetic field generation unit.

### Measurements of the Nernst effect

The unavailability of commercial instruments is another important factor in the slow progress of transverse thermoelectric research. Currently, such measurement can be done only with a custom-built setup, which is available in only a few laboratories in the world. The commercialization of such equipment is highly important so that more researchers can perform the measurement.

As multiple challenges exist in this field, it is therefore creating many opportunities for scientists in research and development. We are hopeful that a close collaborative approach among physicists, chemists, and materials scientists with strong support from theory for materials design can generate rapid progress and development in the field of transverse thermoelectrics.
